# Significance of cerebrovascular reactivity in patients with systemic lupus erythematosus

**DOI:** 10.1186/ar4660

**Published:** 2014-09-18

**Authors:** Jason Lazar, Louis Salciciolli, Elina Malamed, Ellen M Ginzler

**Affiliations:** 1 State University of New York Downstate Medical Center, Brooklyn, NY, USA

## Background

There is growing recognition and concern regarding cognitive dysfunction in patients with systemic lupus erythematosus (SLE). SLE patients have accelerated atherosclerosis, which is known to contribute to cognitive dysfunction in other disease states. Impaired cerebrovascular reactivity (CVR) is a vascular measure linked to cognitive dysfunction, but this has not been well studied in the setting of SLE. The objectives of this study are to determine whether CVR is impaired in patients with SLE and to determine the significance of CVR impairment.

## Methods

Right middle cerebral artery CVR was assessed by the breath holding index (BHI), which combines transcranial Doppler recording and passive breath holding. This technique evaluates changes in middle cerebral artery Doppler velocities upon permissive hypercapnea. We measured the BHI in nine female patients with SLE (age 38 ± 13 years) and 12 age-matched controls. Cognitive function was assessed using the color Stroop block time test (SBT) and the Stroop black/white test (SBWT). The augmentation index, a measure of arterial wave reflection, was assessed by applanation tonometry of the radial artery. Carotid intimal media thickness measurements were obtained with high-resolution ultrasound.

## Results

Completion times for the SBT (38.4 ± 8.7 vs. 29.5 ± 6.6) and the SBWT (29.7 ± 5.4 vs. 22.9 ± 5.8) were significantly higher in SLE patients versus controls (*P *= 0.005 and *P *= 0.004 respectively). On CVR testing, right middle cerebral artery BHI responses were significantly different between SLE and NLS (0.91 ± 1.10 vs. -0.99 ± 1.7, *P *= 0.001). There was a trend towards higher CIMT (0.634 ± 0.130 cm vs. 0.549 ± 0.116 cm, *P *= 0.10) and towards higher AI (27 ± 16% vs. 18 ± 9%, *P *= 0.10) in the SLE group. CVR was significantly correlated with AI (0.68, *P *= 0.003), and a trend with SBT (*r *= 0.42, *P *= 0.07) and CIMT (*r *= 0.45, *P *= 0.08). On multivariate analysis (with age and SLE), SLE was a significant predictor of BHI (*B *= 1.84, *P *= 0.033).

## Conclusions

SLE is associated with impaired CVR. Impaired CVR appears related to higher wave reflection as measured by the AI. This ongoing study will help define the relations between CVR and other vascular parameters as well as potential relations with cognitive dysfunction in SLE patients.

